# Concurrent paraneoplastic dermatomyositis and Sweet syndrome

**DOI:** 10.1016/j.jdcr.2025.09.054

**Published:** 2025-11-07

**Authors:** Alyssa M. Jobe, Ashley T. Ng, Jenna Ruggiero, Kara E. Young, Barbara Wilson, Olushola L. Akinshemoyin Vaughn

**Affiliations:** a Medical College of Wisconsin, Milwaukee, Wisconsin; b Department of Dermatology, Medical College of Wisconsin, Milwaukee, Wisconsin

**Keywords:** dermatomyositis, dermatoses, immune dysregulation, malignancy, paraneoplastic, Sweet syndrome

## Background

Dermatomyositis (DM) is an autoimmune disorder characterized by inflammation of the muscle and skin, thought to be mediated by immunologic dysregulation in genetically susceptible individuals. Sweet syndrome (SS), also known as acute febrile neutrophilic dermatosis, is a rare inflammatory condition marked by edematous cutaneous nodules with dense neutrophilic infiltrate on histology. Each condition is recognized as a paraneoplastic dermatosis, which may manifest before, during, or after the development of an underlying neoplasm. It is uncommon for multiple paraneoplastic dermatoses to occur simultaneously, and only a few cases have documented the coexistence of SS and DM.^1^, ^2^, ^3^ We report a case in which a patient presented with concurrent SS and DM in the setting of an underlying malignancy.

## Report of a case

**Fig 1:**
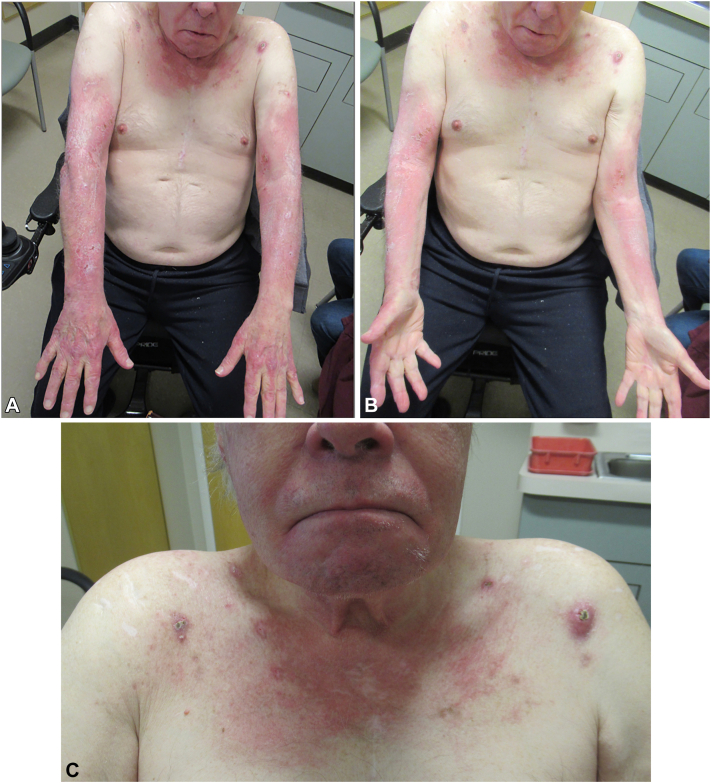
Initial clinical examination. **A/B,** Bright pink patches with minimal scale present on dorsal surfaces on arms with sparing of the shoulders, central chest, and interdigital webbed spaces. **C,** Pink edematous nodules and papules with central crusting scattered on the anterior shoulders and chest.

A 72-year-old male presented to the outpatient dermatology clinic with 4 months of a pruritic rash on the hands, arms, and face that progressed to the upper chest. Over the same period, he reported upper extremity weakness, unintentional weight loss, and dysphagia. Examination was significant for diffuse, bright pink patches with minimal overlying scale on the central upper chest, neck, bilateral distal upper arms, dorsal forearms, and dorsal hands in a photodistributed pattern (Fig 1). Additionally, there were a few pink edematous nodules with central crusting scattered on the anterior shoulders and chest. Laboratory workup was performed for underlying connective tissue disease and treatment with fluocinonide 0.05% ointment twice daily was recommended, with moderate improvement in the pruritic rash; however, due to worsening dysphagia and weakness, he was admitted for further evaluation.

**Fig 2:**
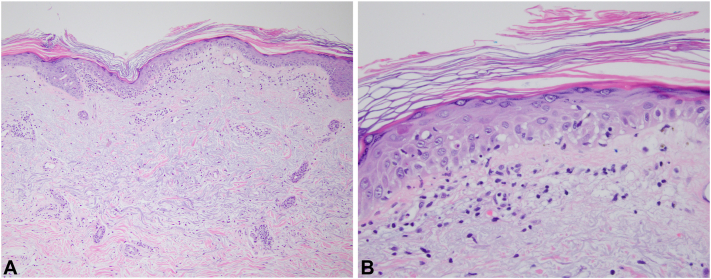
Histopathologic examination of the cutaneous patch on the left forearm with hematoxylin-eosin staining showed mild hyperkeratosis with vacuolar interface alteration and a sparse superficial perivascular lymphocytic infiltrate (**A,** 10×, **B,** 40×) (not pictured here; however, direct immunofluorescence studies were performed and showed nonspecific findings of weak linear deposits of IgG and fibrinogen along the basement membrane zone. Granular deposits of C3 were noted as well as some IgM cytoid bodies).

**Fig 3:**
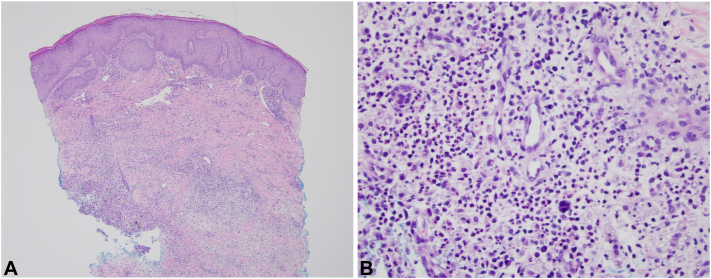
Histopathologic examination of a nodule on the left shoulder with hematoxylin-eosin staining showed a diffuse inflammatory infiltrate within the dermis (**A,** 4×) with abundant neutrophils (**B,** 40×) and mild dermal edema.

Laboratory workup was notable for mildly elevated creatine kinase (260), aspartate aminotransferase (62), and aldolase (9). Complete blood count with differential was unremarkable. Erythrocyte sedimentation rate (81 mm/h) and C-reactive protein (74.1 mg/L) were elevated. Serological tests revealed negative antinuclear, anti-Jo-1, and anti-cN1a antibodies. Myositis antibody panel demonstrated elevated transcriptional intermediary factor 1 gamma autoantibody (150). Punch biopsy of the left forearm showed vacuolar interface dermatitis with a histologic differential diagnosis of a connective tissue process or drug eruption (Fig 2). Direct immunofluorescence revealed a mixed and nonspecific pattern. A second punch biopsy of a nodule on the anterior shoulder was notable for a diffuse inflammatory infiltrate with abundant neutrophils in the dermis, mild dermal edema, and negative special stains for microorganisms, supportive of a neutrophilic dermatosis (Fig 3). Given the atypical mild elevation of muscle enzymes, the patient underwent a confirmatory muscle biopsy which demonstrated an inflammatory myopathy consistent with DM. Additional workup for an underlying malignancy ultimately revealed a bladder mass on imaging, and subsequent transurethral resection of bladder tumor revealed invasive urothelial carcinoma with high-grade squamous, neuroendocrine, and glandular differentiation.

Collectively, his clinical, histopathologic, laboratory, and imaging findings were consistent with a concurrent presentation of DM and SS in the setting of underlying malignancy.

This patient was treated with 250 mg intravenous methylprednisolone daily for three days, then prednisone taper, intravenous immunoglobulin (2 doses of 1 mg/kg over 2 days for 3 total rounds), and 15 mg weekly methotrexate with substantial improvement in cutaneous symptoms and weakness. He planned to undergo a radical cystectomy with ileal conduit for his urothelial carcinoma. However, after discharge, the patient experienced a relapse of cutaneous symptoms and weakness in the setting of weaning systemic corticosteroids and a delay in intravenous immunoglobulin infusion. He unfortunately passed away shortly thereafter.

## Discussion

Malignancy has been identified in approximately 14.8% of DM cases, most often involving the gastrointestinal tract, lungs, nasopharynx, and ovaries, with elevated TIF-1, as seen in this case, further raising concern for an underlying neoplastic trigger.^1^^,^^4^^,^^5^ The pathogenesis of DM in the paraneoplastic setting involves both immune and vasculopathic mechanisms. T cell–mediated myocytotoxicity contributes to muscle fiber damage manifesting as weakness, while complement-mediated destruction of endomysial capillary endothelium leads to microangiopathy and tissue ischemia.^6^

SS may be classified as paraneoplastic, para-inflammatory (associated with underlying autoimmune conditions such as systemic lupus erythematosus or inflammatory bowel disease), drug-induced, or idiopathic.^1^^,^^7^ About 10% to 20% of SS cases are paraneoplastic, and of identified malignancies, 85% are hematologic, including acute myeloid leukemia, chronic myeloid leukemia, and multiple myeloma.^1^ Studies have shown that the production of inflammatory cytokines including interleukin (IL) 1, IL-3, IL-6, IL-8, granulocyte colony stimulating factor, and granulocyte macrophage colony stimulating factor likely plays a role in paraneoplastic SS.^8^

The concurrent presentation of DM and SS, while rare, may reflect shared pathophysiologic mechanisms in the context of an underlying malignancy. Inflammatory mediators may be secreted directly by tumors or indirectly stimulated through paraneoplastic immune activation, providing a possible mechanistic link between the 2 dermatoses. Additionally, overlapping treatments, including immunosuppressants, suggest that while the underlying immune pathways may diverge in specific cellular mediators, the broader inflammatory response remains susceptible to shared therapeutic strategies. Recognizing the potential for concurrent paraneoplastic dermatoses not only broadens our understanding of their shared immunopathogenesis but also highlights the importance of a comprehensive malignancy screening to ensure timely diagnosis and treatment.

To our knowledge, there are only 2 other documented cases of concurrent DM and SS. One case identified a patient with known DM who developed SS after completion of a prednisone taper.^2^ Notably, no underlying malignancy was identified, suggesting an immune-mediated etiology rather than paraneoplastic. The second case described the development of SS in a patient with a long history of poorly controlled DM, non-Hodgkin lymphoma in remission, and recent severe infection.^3^ In this context, it is challenging to determine the precise trigger of SS. These cases highlight the complex interplay between immune dysregulation, malignancy, and external factors in the pathogenesis of paraneoplastic dermatoses. Our case further illustrates this complex relationship as it is unclear whether our patient’s SS represents an independent entity, a paraneoplastic manifestation parallel to DM, or secondary to the autoimmune process of DM.

The presentation of DM and SS should prompt a thorough evaluation for underlying malignancy given their established associations with paraneoplastic processes. This case underscores the importance of maintaining a broad differential, particularly when multiple paraneoplastic dermatoses coexist, given the uncommon genitourinary malignancy. Early identification of an occult neoplasm can significantly alter prognosis and guide both dermatologic and oncologic management.

## Conflicts of interest

None disclosed.
